# The two authentic methionine aminopeptidase genes are differentially expressed in *Bacillus subtilis*

**DOI:** 10.1186/1471-2180-5-57

**Published:** 2005-10-05

**Authors:** CongHui You, HongYan Lu, Agnieszka Sekowska, Gang Fang, YiPing Wang, Anne-Marie Gilles, Antoine Danchin

**Affiliations:** 1 National Laboratory of Protein Engineering and Plant Genetic Engineering, The College of Life Sciences, Peking University, 100871, Beijing, P.R. China; 2 Institut Pasteur, Unité de Génétique des Génomes Bactériens, 28 rue du Docteur Roux, 75724 Paris Cedex 15, France; 3 HKU-Pasteur Research Centre, Dexter H. C. Man Building, 8 Sassoon Road Pokfulam, Hong Kong, S.A.R., P.R. China; 4 CEA Saclay, Laboratoire Stress Oxydants et Cancer, DSV/DBJC/SBMS, Bat 142, 91191 Gif sur Yvette Cedex, France

## Abstract

**Background:**

Two putative methionine aminopeptidase genes, *map *(essential) and *yflG *(non-essential), were identified in the genome sequence of *Bacillus subtilis*. We investigated whether they can function as methionine aminopeptidases and further explored possible reasons for their essentiality or dispensability in *B. subtilis*.

**Results:**

*In silico *analysis of MAP evolution uncovered a coordinated pattern of MAP and deformylase that did not correlate with the pattern of 16S RNA evolution. Biochemical assays showed that both MAP (MAP_Bs) and YflG (YflG_Bs) from *B. subtilis *overproduced in *Escherichia coli *and obtained as pure proteins exhibited a methionine aminopeptidase activity *in vitro*. Compared with MAP_Bs, YflG_Bs was approximately two orders of magnitude more efficient when assayed on synthetic peptide substrates. Both *map *and *yflG *genes expressed in multi-copy plasmids could complement the function of a defective *map *gene in the chromosomes of both *E. coli *and *B. subtilis*. In contrast, *lacZ *gene transcriptional fusions showed that the promoter activity of *map *was 50 to 100-fold higher than that of *yflG*. Primer extension analysis detected the transcription start site of the *yflG *promoter. Further work identified that YvoA acted as a possible weak repressor of *yflG *expression in *B. subtilis in vivo*.

**Conclusion:**

Both MAP_Bs and YflG_Bs are functional methionine aminopeptidases *in vitro *and *in vivo*. The high expression level of *map *and low expression level of *yflG *may account for their essentiality and dispensality in *B*. *subtilis*, respectively, when cells are grown under laboratory conditions. Their difference in activity on synthetic substrates suggests that they have different protein targets *in vivo*.

## Background

Ribosome-mediated protein synthesis is always initiated with either methionine (in eukaryotes) or N-formylmethionine (in prokaryotes and eukaryotic organelles) [1]. However, after removal of the N-formyl group from the polypeptide by peptide deformylase (DEF, EC 3.5.1.88), the N-terminal methionine of a large number of proteins is cleaved by methionine aminopeptidase (MAP, EC 3.4.11.18) [2,3]. The efficiency of removal of the initiator methionine is defined by a highly conserved local substrate specificity, which is determined by both methionine and its adjacent residue. MAP hydrolytically removes the N-terminal methionine only when the penultimate residue bears a small and uncharged side chain (Gly, Ala, Ser, Thr, Pro, Val, or Cys) [4-9], and residues downstream of the penultimate residue have little impact on the reaction. All MAPs studied to date were reported to be cobalt-dependent metalloproteases [3]. However, some reports showed that MAPs exhibited activity in the presence of other divalent ions, such as Zn(II), Fe(II) or Mn(II) [10-12]. According to their sequence homology, MAPs are grouped into two subtypes, type I and type II [13,14]. An insertion of approximately 60 residues in the C-terminal domain of type II methionine aminopeptidases is the only difference between the two types [14]. Eukaryotes possess at least two *map *genes, of both type I and type II [3], while there is only one *map *gene in most prokaryotic genomes, either type I (Archaea) or type II (Bacteria) [15].

MAP is distributed throughout living organisms, where it plays an important physiological role. The deletion of the single *map *gene in prokaryotes such as *Escherichia coli *[16] or *Salmonella typhimurium *[17] is lethal. In yeast, the deletion of any one of the two *map *genes (type I or type II) causes a slow growth phenotype, while deletion of both genes is lethal [18]. Why might MAP activity have an essential role in living cells? Previous studies showed that many proteins needed to have their N-terminal methionine removed to have normal biological activity, proper subcellular localization and eventual degradation (reviewed in [19]). As a result, it is possible that MAP is essential because of the essentiality of its downstream targets. Besides essentiality, MAP changes the dynamics of the sulfur containing metabolites, which may have an important role in the homeostasis of the cell.

In general, there is only one *map *gene in the genome of prokaryotes, with the exception of a cyanobacteria strain, which has three functional methionine aminopeptidases [20]. Surprisingly, in the genomes of *Bacilli*, two or even more putative MAP genes can be detected by sequence alignment. In *B. subtilis*, two putative genes responsible for MAP activity, *map *and *yflG*, can be identified [21]. Kobayashi *et al*. [22] reported that *map *is an essential gene in *B. subtilis *while *yflG *is not, an apparent paradox if *yflG *codes for a methionine aminopeptidase. In an attempt to better understand the functional role of MAP, we studied the evolutionary trend of MAPs *in silico *and demonstrated that both the *map *and *yflG *genes from *B. subtilis *code for methionine aminopeptidases *in vitro *and *in vivo*; furthermore, the finding of a high expression level for *map *in parallel with a low expression of *yflG *in *B. subtilis*, may account for their essentiality and dispensability, respectively.

## Results

### Sequence alignment and evolutionary pattern analyses

**Figure 1:**
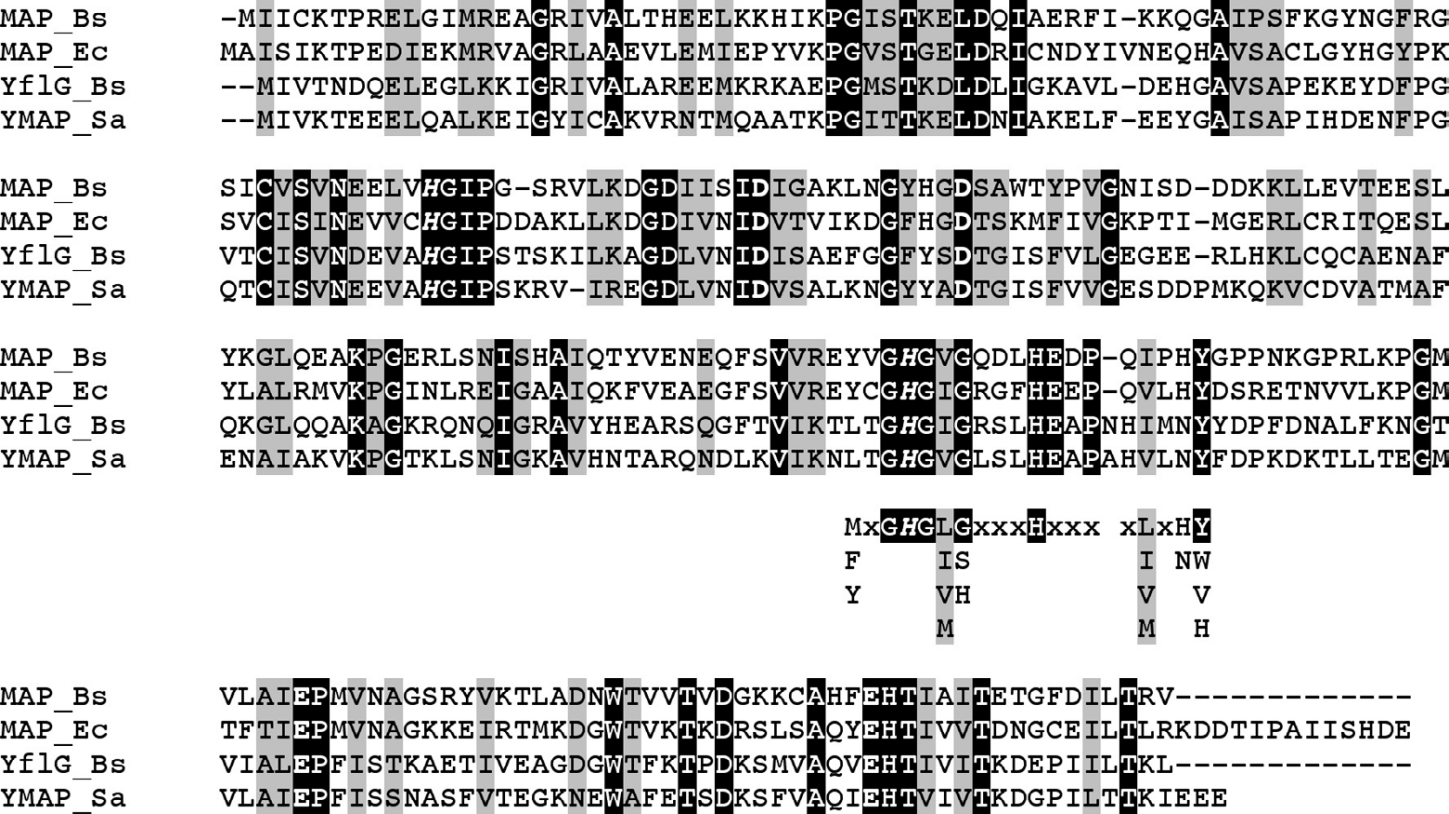
**Sequences alignment between MAP from *E. coli*, *B. subtilis*, and *S. aureus*. **The first line shows the protein sequence of MAP from *B. subtilis *(MAP_Bs), the second shows MAP from *E. coli *(MAP_Ec), the third shows YflG from *B. subtilis *(YflG_Bs) and the last shows YflG/MAP from *Staphylococcus aureus *(YMAP_Sa). Identical amino acids are shadowed in dark and conservative amino acids are shadowed in grey. The highlighted residues stand for those conserved amino acids important for MAP activity (bold and italic residues are conservative basic ones, while bold only are acidic ones). [MFY]-x-G-H-G-[LIVMC]-[GSH]-x(3)-H-x(4)-[LIVM]-x-[HN]-[YWVH] (where H is a cobalt ligand) is the conserved motif within the protein sequences (placed under the alignment).

In the genome sequence of *B. subtilis*, two genes could code for methionine aminopeptidases, *map *and *yflG*, respectively [21] (Figure 1). The sequence similarity and identity between *E. coli *MAP (MAP_Ec) and *B. subtilis *MAP (MAP_Bs) is 65% and 46%, respectively. Similarity between MAP_Ec and YflG from *B. subtilis *(YflG_Bs) is 55% and their identity is 34% and similarity between MAP_Bs and YflG_Bs is 58% and their identity is 36%. However, the sequence of YflG_Bs differs slightly from the PROSITE consensus for type I MAPs (PS00680), which is [MFY]-x-G-H-G- [LIVMC]-[GSH]-x(3)-H-x(4)-[LIVM]-x-[HN]-[YWVH]. In particular it displays one extra residue next to the conserved GHG metal-binding motif. Interestingly, MAP_Bs is more similar to MAP_Ec and YflG_Bs is more similar to the only putative methionine aminopeptidase of *Staphylococcus aureus *(labelled as "YMAP" in Figure 1). Overall the proteins are very similar in their physico-chemical properties (they have closely similar length and are slightly acidic). Moreover, as shown in Figure 1, the metal (presumably cobalt) binding site (highlighted residues) is conserved. It was therefore important to investigate whether both proteins had methionine aminopeptidase activity.

**Figure 2:**
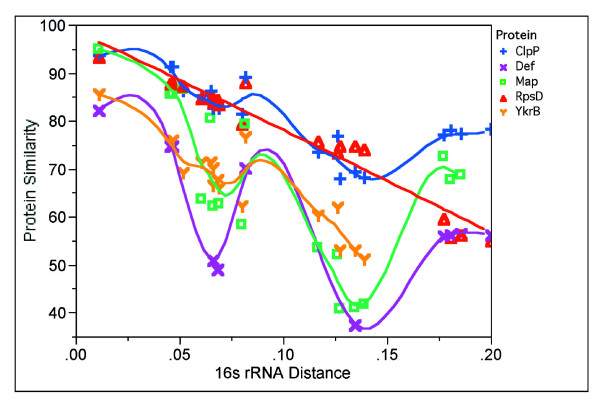
**Evolutionary trend analyses of MAP, ClpP, Def, RpsD and YkrB. **Map, ClpP, Def, RpsD and YkrB were picked up from *B. subtilis*. Their BBHs from other Firmicutes were detected. Their protein divergence (sequence similarity divided by sequence length difference) in *B. subtilis *and their orthologs from other Firmicutes were compared with bacterial evolutionary distance individually (measured by 16S rRNA evolution).

This difference in evolution of proteins considered as essential in related organisms (Firmicutes) is intriguing, and we explored the evolution pattern of *map *in a study meant to identify persistent genes in bacteria [23]. When matched with the evolution of 16S RNA, the *map *gene does not follow a linear course of evolution, as do most ribosomal proteins [23], but shows an erratic pattern (Figure 2). This suggests that MAP might be involved in different biological processes besides its role as methionine aminopeptidase according to the organisms. This prompted us to explore the evolution of MAP with proteins that might be functionally related, using the criteria we used to identify persistent genes. We defined persistent genes as the genes present as orthologs in more than 85% of the genomes of a clade (here 26 Firmicutes), after removing obligatory endosymbionts from the list (see Methods). The protein divergence (sequence similarity divided by sequence length difference) of MAP in *B. subtilis *and its orthologs from other Firmicutes were compared with bacterial evolutionary distance (measured by 16S rRNA distance). Similar analyses were performed with a general protease, ClpP, DEF, ribosomal protein S4, RpsD and YkrB, a second deformylase present in *B. subtilis *(Figure 2). Interestingly, in contrast to the expected evolutionary pattern of RpsD, MAP exhibited the same erratic evolutionary way as Def and YkrB, the two functional deformylases in *B. subtilis*, suggesting co-evolution of methionine aminopeptidase and deformylase.

### Enzyme activity *in vitro*

It is often recognized that gene function identification solely based on sequence comparisons could be misleading [24,25]. Therefore, we first determined whether *map *and *yflG *were authentic MAP genes by overproducing the proteins in *E. coli *and characterizing their enzymatic activity. Both products of *map *and *yflG *genes were purified to more than 90% homogeneity. Mass spectrometry showed that the molecular mass of MAP_Bs and YflG_Bs was 27409.17 ± 0.92 Da and 27209.35 ± 2.14 Da, respectively. Interestingly, both proteins purified from *E. coli *retained their initial methionine, consistent with the nature of the second residue, isoleucine in both cases.

As seen in Table 1, both enzymes exhibited a methionine aminopeptidase activity *in vitro *with the synthetic peptide substrates tested. They differed widely, however, in the extent of their activity: the specific activity of YflG_Bs was about 90- and 20-fold higher than that of MAP_Bs with the tetrapeptide MGMM and tripeptide MAS as the substrates, respectively. Both enzymes preferred the tetrapeptide MGMM as the substrate. With MAS and MG as the substrates, MAP_Bs activity was 76.9% and 46.2% of that with MGMM as the substrate, respectively, while YflG_Bs retained only 17.7% and 0.6% relative activity, respectively, with these substrates.

**Figure 3:**
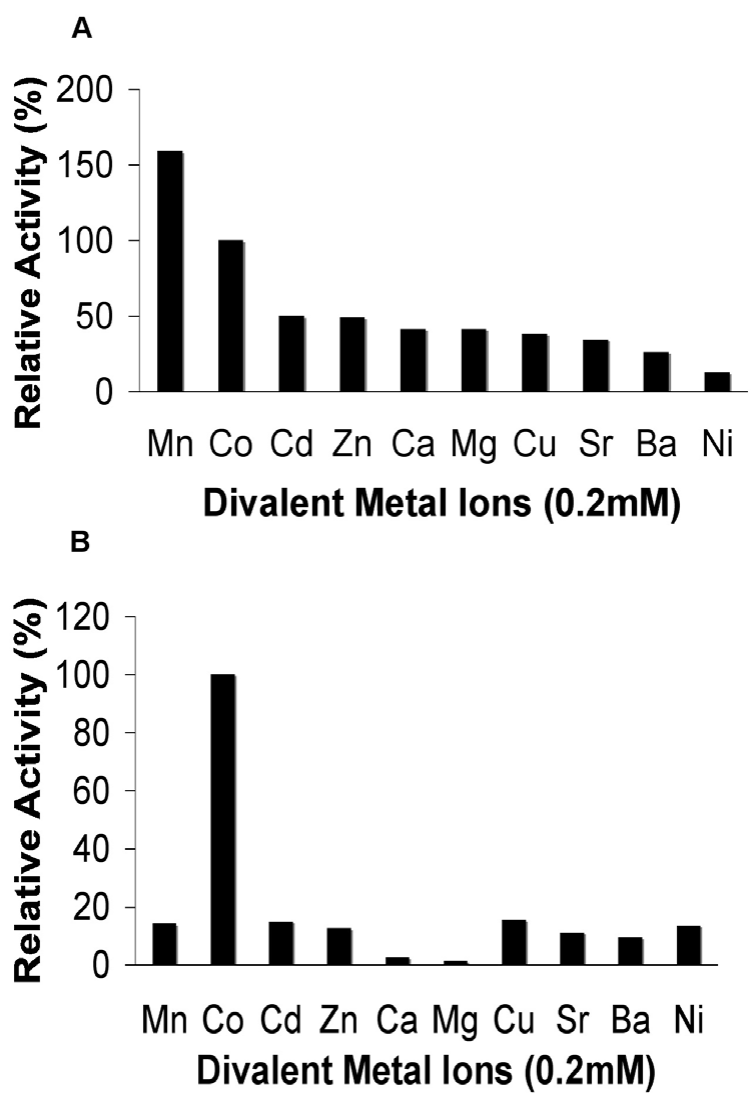
**Relative activity of (A) MAP_Bs and (B) YflG_Bs in the present of different ions. **MGMM (4 mM) was used as the substrate. All ions were tested at 0.2 mM concentration. Each of their relative activity was defined as 100% in the presence of Co^2+ ^(MAP_Bs: 2.6 U/mg; YflG_Bs: 232.0 U/mg).

The effect of divalent ions (0.2 mM) on enzyme activity was investigated (Figure 3). MAP_Bs had significant activity with both Mn^2+ ^and Co^2+^. MAP_Bs retained appreciable activity (26–50%) after replacement of Co^2+ ^with Cd^2+^, Zn^2+^, Ca^2+^, Mg^2+^, Cu^2+^, Sr^2+ ^and Ba^2+^, but not Ni^2+^. In contrast, YflG_Bs showed a strong preference for Co^2+ ^with a decrease in activity to below 20% with any other divalent ions tested.

### Subcellular localization

**Figure 4:**
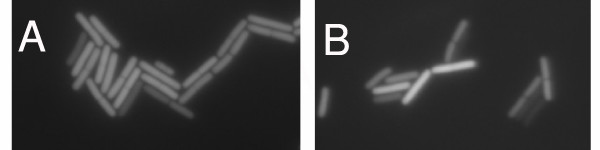
**Localization of GFP-MAP_Bs and GFP-YflG_Bs fusions. **A. Localization of GFP-MAP_Bs in strain BSIP8001 grown in minimal medium with 0.5% xylose (w/v); B. Localization of GFP-YflG_Bs in strain BSIP8002 grown in minimal medium with 1% xylose (w/v).

To investigate whether MAP_Bs and YflG_Bs had any difference in their subcellular localization, we constructed N-terminal green fluorescent protein (GFP) fusions of both proteins. As previously reported, the catalytic domain of the methionine aminopeptidase is located in the C-terminal part of the polypeptide chain [20,26-29], therefore N-terminal GFP fusions of both proteins were not expected to interfere with their functions. In addition, we also constructed C-terminal GFP fusions of both MAP_Bs and YflG_Bs, but because of the possible degradation of the fusion proteins *in vivo *or some unknown reasons, we failed to detect any fluorescence in either case (data not shown). Cells from strains BSIP8001 (MAP::GFP fusion) and BSIP8002 (YflG::GFP fusion) in the mid-exponential growth phase were collected and visualized by fluorescence microscopy, respectively. Both GFP-MAP_Bs (Figure 4A) and GFP-YflG_Bs (Figure 4B) proteins distributed evenly all over the cells. As was expected, the intensity of the fluorescence of both proteins increased with the increasing concentration of xylose used to induce expression of the GFP fusions (0.1%- 1% (w/v)) (data not shown).

### MAP-defective mutants are rescued by MAP_Bs or YflG_Bs supplied in trans

**Figure 5:**
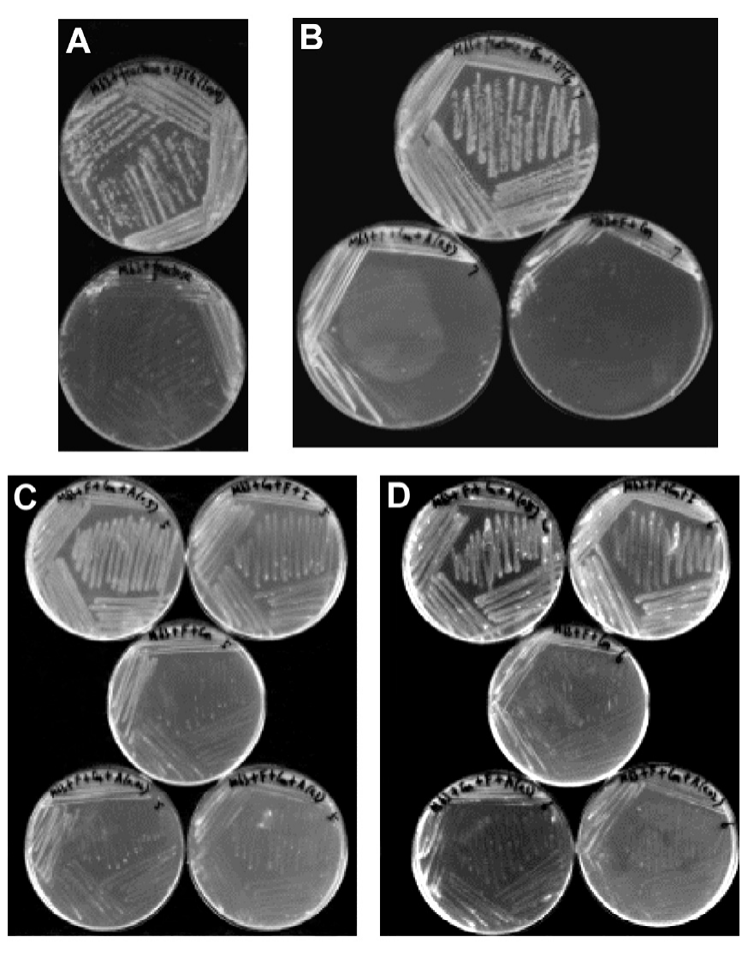
**Genetic rescue experiments. **The bacteria were streaked on M63 minimal solid medium and the plates were incubated at 37°C overnight. The concentration of IPTG was 1 mM. The order of the plates in each picture described below is from left to right and from top to bottom. A. EM9 host strain on M63 with IPTG; EM9 on M63 without IPTG. B. EM9 strain with pBAD vector alone (ECHP1007) on M63 with IPTG; ECHP1007 on M63 with arabinose (0.5% (W/V)); ECHP1007 on M63 without IPTG and arabinose. C. EM9 strain caring pBAD *map *vector (ECHP1005) on M63 with arabinose (0.5% (W/V)); ECHP1005 on M63 with IPTG; ECHP1005 on M63 without IPTG; ECHP1005 on M63 with arabinose (0.02% (W/V)); ECHP1005 on M63 with arabinose (0.1% (W/V)). D. EM9 strain caring pBAD *yflG *vector (ECHP1006) on M63 with arabinose (0.5% (W/V)); ECHP1006 on M63 with IPTG; ECHP1006 on M63 without IPTG; ECHP1006 on M63 with arabinose (0.1% (W/V)); ECHP1006 on M63 with arabinose (0.02% (W/V)).

The *E. coli *mutant EM9 contains an engineered *map *gene, whose expression is under the control of isopropyl β-D-thiogalactoside (IPTG), so that the strain cannot grow unless IPTG is added to the growth medium [16]. Using the replicative vector pBAD, which holds an arabinose-inducible promoter, the *map *and *yflG *genes from *B. subtilis *were individually introduced into strain EM9. As shown in Figure 5, the transformants could complement the *map *defect when IPTG was omitted (leading to a MAP deficiency) if a high concentration of arabinose was supplied, in the case of plasmids carrying either *B. subtilis map *or *yflG *genes. Lower concentrations of arabinose only partially supported growth. This demonstrated that both *map *and *yflG *from *B. subtilis *can supply the MAP function to *E. coli in vivo*.

In *B. subtilis*, *map *is an essential gene, while *yflG *is not [22]. However, with a back-up *map *or *yflG *copy under the control of IPTG provided in multi-copy plasmid pDG148 (30–50 copies using the pUB110 replicon from *B. subtilis *[30]), *map*::*lacZ *disruptants in the chromosome of *B. subtilis *were obtained (BSHP7046 and BSHP7037). Nevertheless, as witnessed in both cases by the systematic formation of variegated blue white streaked colonies on X-gal plates, the clones were extremely unstable, leading to the loss of transformants after a few generations (data not shown).

### *map *expression is considerably higher than *yflG *expression

The fact that *map *is essential while *yflG *is not [22], together with the ability of *yflG *gene to rescue MAP_Bs function when supplied at a high level, points to substantial differences in the expression level of the two genes. To test this possibility, the expression of *map *and *yflG *genes in *B. subtilis in vivo *was studied using *lacZ *as the reporter gene.

**Figure 6:**
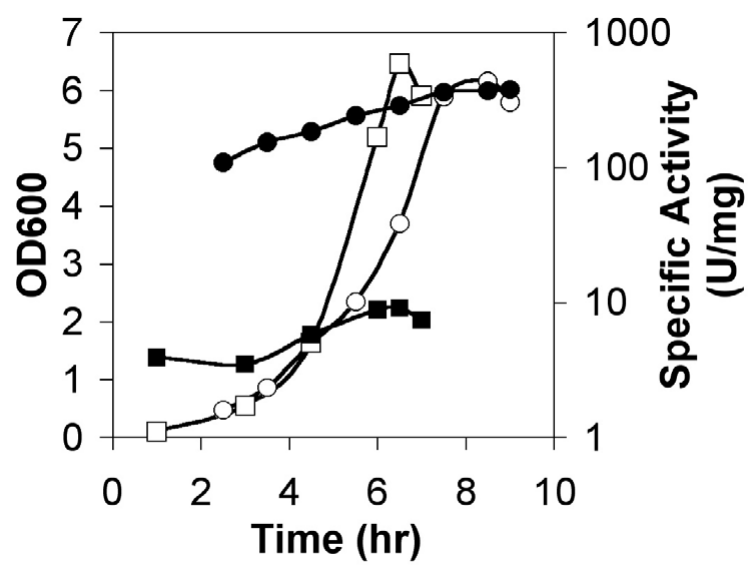
**Expression of *map *and *yflG lacZ *transcriptional fusions. **β-galactosidase expression of *map *and *yflG lacZ *transcriptional fusion during the growth curve of strains BSHP7042 and BFS4611 in minimal medium. OD_600 _(○) and β-galactosidase activity (●) were measured for strain BSHP7042 with *map lacZ *transcription fusion. OD_600 _(□) and β-galactosidase activity (■) were measured for strain BFS4611 with *yflG lacZ *transcription fusion.

Strains BSHP7042 and BFS4611 were grown in minimal medium and were used to monitor the expression of *map *and *yflG*, respectively. The *map *gene showed the higher promoter activity (Figure 6). Its activity increased gradually during the log phase of growth and a nearly four-fold increase was detected (109–379 U/mg) between early exponential and stationary growth phases. In contrast, the *yflG *gene showed very low activity under all the conditions tested (4–8 U/mg) (Figure 6). In summary, the *map *gene promoter showed 50 to 100-fold higher activity than that of *yflG *using *lacZ *as the reporter gene.

### Promoter localization of the *yflG *gene

**Figure 7:**
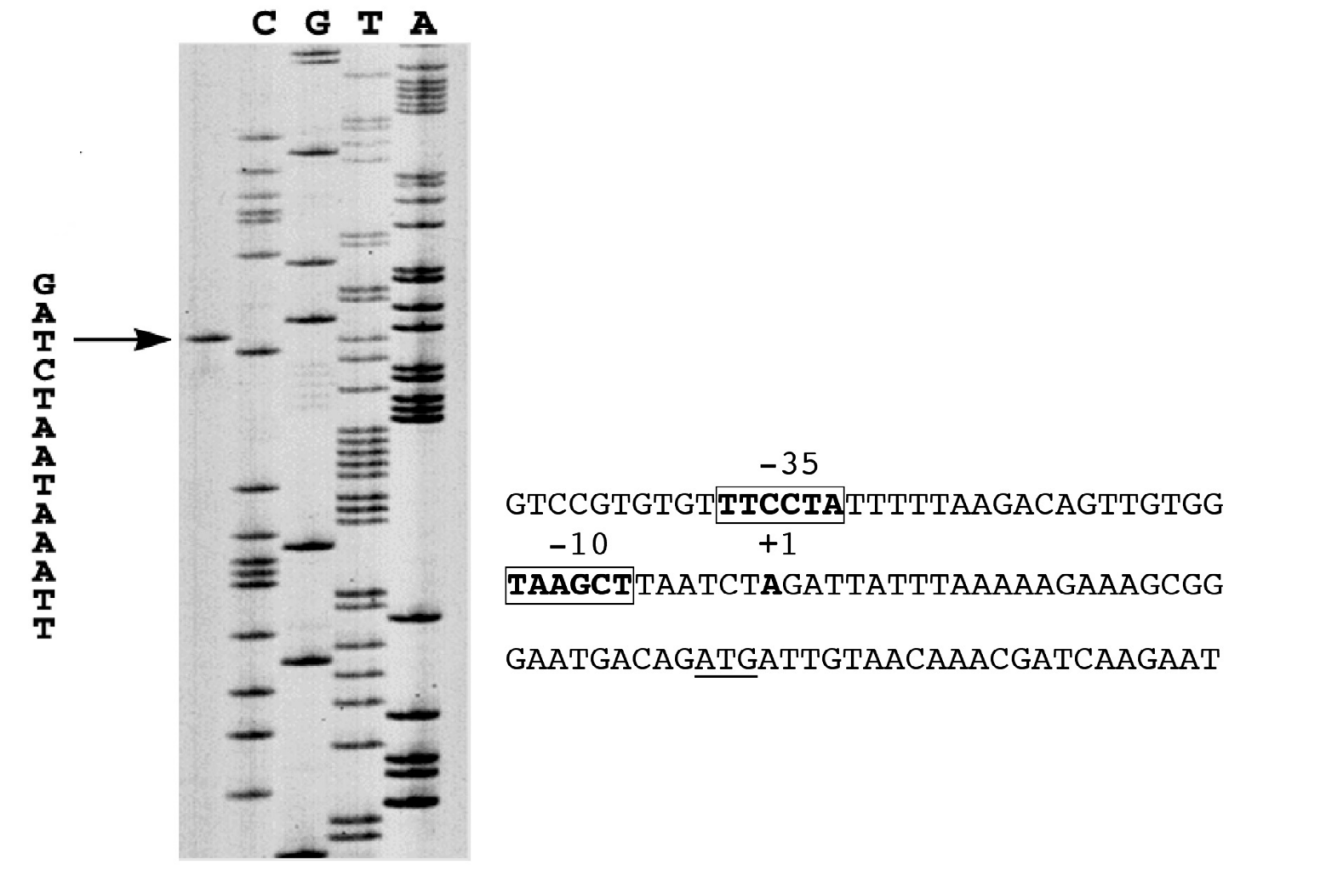
**Identification of the transcription start point of the *yflG *promoter. **Primer extension and sequencing reaction were performed with the same primer. The size of the extended product is compared to a DNA-sequencing ladder of the corresponding region. An arrow indicates the +1 site. Predicted -10 and -35 regions are in bold and boxed; the experimental transcription start site is in bold; the translational start site is underlined.

In the *B. subtilis *genome, the *yflG *gene is located upstream of the *yflH *gene, followed by a putative Rho-independent transcription terminator. RT-PCR experiments demonstrated that *yflG *and *yflH *belonged to a common transcription unit, making an operon (data not shown). Furthermore, *lacZ *transcription fusion with *yflH *gene (strain BSHP7043) showed the same activity changes as that of *yflG *gene during all the conditions tested (data not shown), substantiating the RT-PCR results. In order to identify the promoter of the *yflG*-*yflH *operon, we carried out primer extension analysis. Two primers complementary to two different regions inside the *yflG *gene were used yielding identification of the same transcription start point. As shown in Figure 7, the start is located 31 nt upstream of the ATG translation start codon and regions weakly similar to consensus -35 (TTCCTA) and -10 (TAAGCT) regions are found upstream of this start point, separated by 18 nt, in an AT-rich region. It is difficult at this point to correlate the structure of this promoter with the poor expression of *yflG *in all conditions tested.

### YvoA showed a slight repression effect on *yflG *expression

**Figure 8:**
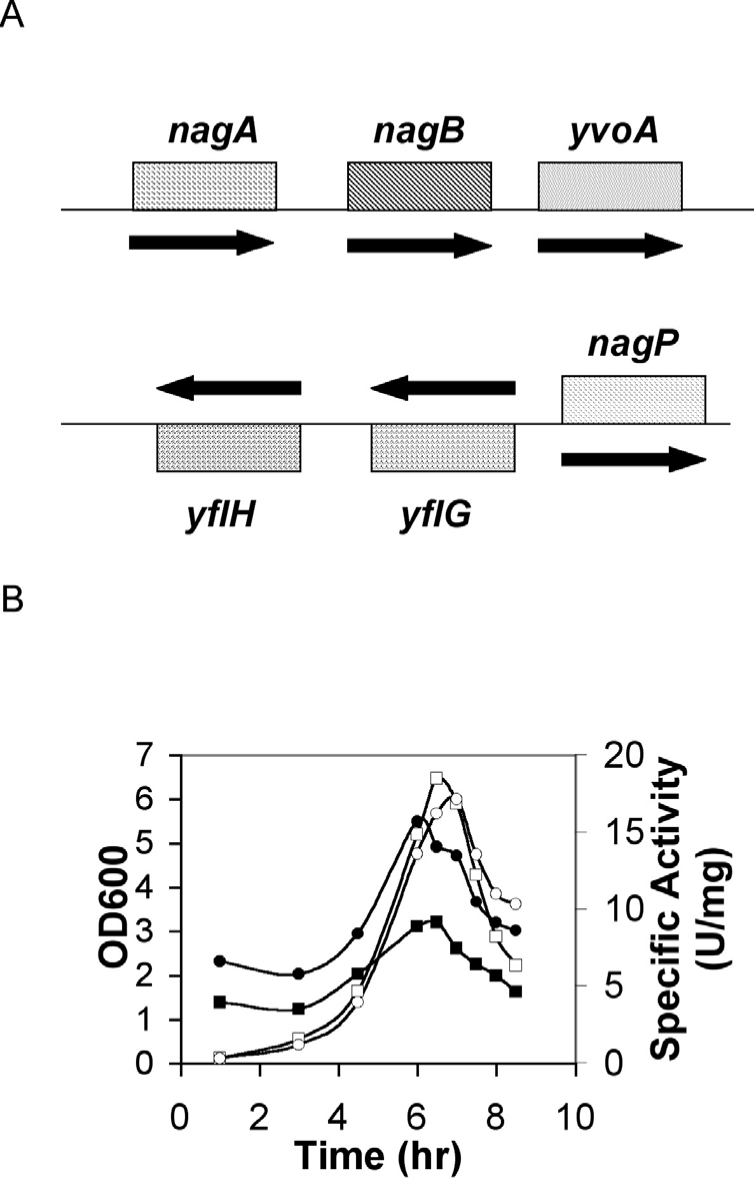
**Expression of *yflG lacZ *transcriptional fusions in *yvoA *disrupted strain. **A. gene organization around *yflG *and *yvoA*. The arrows indicate the direction of gene transcription; B. β-galactosidase expression *yflG lacZ *transcriptional fusion in *yvoA *disrupted strain BSIP8004 and *yvoA *wild type strain BFS4611 grown in minimal medium. OD_600 _(○) and β-galactosidase activity (●) were measured for strain BSIP8004. OD_600 _(□) and β-galactosidase activity (■) were measured for strain BSIP8004 and strain BFS4611.

As the expression of *yflG *in *B. subtilis in vivo *was very low, we endeavoured to uncover conditions to enhance its expression. No significant difference was found when the carbon, nitrogen and sulfur sources were changed, and no difference was found in conditions of sporulation or germination (data not shown). As genes proximal in the chromosome often code for proteins with related functions, the neighbourhood of *yflG *was analysed. Gene *nagP*, which encodes a putative phospho*enol*pyruvate-dependent transport system N-acetylglucosamine-specific enzyme IICB component, is located upstream of *yflG *and transcribed divergently (Figure 8A). It could share common control elements. Exploring the corresponding intergenic region with a sliding window 19 nt-long we uncovered sequence AATTGGTATAGATCACTAG which has a very significant counterpart (AGCTGGTCTAGATCACTAG) upstream of the *nagAB *operon, encoding two N-acetylglucosamine metabolic genes and a putative transcriptional regulator (GntR family) gene, *yvoA *(Figure 8A). This prompted us to investigate whether YvoA was a possible regulator for *yflG*. Using a *lacZ *fusion, we monitored the expression of *yflG *in a *yvoA*-disrupted strain, BSIP8004.

As shown in Figure 8B, the promoter of *yflG *showed a low but consistently higher activity in the *yvoA *gene disrupted strain BSIP8004 when compared with the wild type parent BFS4611 (two-fold higher activity). This suggested that YvoA could contribute to the regulation of *yflG *expression.

## Discussion

In prokaryotes, it is usually accepted that there is only one gene responsible for methionine aminopeptidase. However, the *B. subtilis *genome program predicted the existence of two methionine aminopeptidase genes, *map *and *yflG *[21]. We have shown that both MAP_Bs and YflG_Bs are functional methionine aminopeptidases *in vitro *(Table 1 and Figure 3). Furthermore, gene rescue experiments showed that both *map *and *yflG *genes expressed on replicative plasmids could supply the MAP function in *E. coli *and *B. subtilis*, demonstrating that both MAP_Bs and YflG_Bs are functional methionine aminopeptidases *in vivo *(Figure 5). Interestingly, Kobayashi *et al*. [22] reported that disruption of the *map *gene in *B. subtilis *is lethal, while the deletion of *yflG *is not. In the present study, we obtained *B. subtilis map *mutants when *map *or *yflG *genes were expressed in multi-copy replicative plasmids under the control of an IPTG induced promoter (strains BSHP7046 and BSHP7037). This observation suggests that the single copy of the *yflG *gene expressed in the chromosome could not supply the MAP function in *B. subtilis*, substantiating that under our conditions its expression is extremely low, making MAP the only significant methionine aminopeptidase available. This is corroborated by the *lacZ *fusion studies showing that the *map *gene is expressed some 50–100-fold higher than *yflG *under all conditions tested. Despite the weak expression of *yflG*, however, we detected a sigma A promoter using primer extension (Figure 7). We further showed that this expression is modulated by YvoA (Figure 8), suggesting a possible connection between N-acetylglucosamine metabolism and methionine aminopeptidase. In this respect it is worth noting that in *E. coli *glucosamine-6-phosphate deaminase activity is modulated by the amino-terminal methionine of the enzyme [31]. We tried several growth conditions involving N-acetylglucosamine as a carbon or nitrogen source, or both, but did not find any modulation of YflG activity (data not shown). We also explored expression of *yflG*::*lacZ *fusions during sporulation and germination. No enhancement was observed (data not shown). As a consequence, we surmise that the high expression level of *map *accounts for its essentiality in *B*. *subtilis.*

The published MAP_Ec activity [6,11] is about 5–10 times higher than that of MAP_Bs in our assay conditions. This may be related to the observation that the *map *gene in *E. coli *does not belong to a highly expressed ribosome protein operon, in contrast to the situation in *B. subtilis *[32]. As a result, the relative expression level of *map *in *E. coli *might be lower than that of the *map *gene in *B. subtilis*. For still unknown reasons it might be impossible for the *yflG *gene to function as the main methionine aminopeptidase in *B. subtilis in vivo *in spite of the high activity of YflG_Bs *in vitro. *As shown in Table 2, the enzyme activity of MAPs in crude cell extracts of *E. coli *(BL5), *B. subtilis *(168) and *yflG *disrupted *B. subtilis *strain (BFS4611) showed not much difference *in vitro *under all the condition tested. Different expression levels of *map *genes in a genome carrying more than one methionine aminopeptidase genes have been previously suggested to be the main reason for their different physiological roles *in vivo*, but no direct evidence was provided [20,33]. Methionine aminopeptidases *in vivo *are active on proteins, i.e. on complex polypeptides [34,35] and not on the short peptides in our *in vitro *assays. It could well be that specific targets require a specific aminopeptidase. A similar conclusion holds for other apparently paralogous genes. For example, there are also two functional peptide deformylases in *B. subtilis*, but only one is probably the predominant deformylase with concomitant high gene expression [36].

The finding of the co-evolutionary parallel trend of methionine aminopeptidase and deformylase in Firmicutes (Figure 2) is consistent with functional relationship *in vivo*, which correlates well with the fact that deformylase removes all N-formyl groups as a prerequisite for the subsequent function of MAP [33]. This co-evolution may be the signature of protein-protein interactions [37,38], an intriguing conjecture, which seems to be supported by the consistent duplication of deformylases in genomes with more than one methionine aminopeptidase (Table 3).

## Conclusion

We proved that both MAP_Bs and YflG_Bs are functional methionine aminopeptidases in *B. subtilis *and we suggested that *map *gene is essential because of its high expression level, while *yflG *is nonessential possibly because of its low expression level making that it can not take over or compensate the function of *map *when *map *is not expressed, or because of specific targets dedicated to only one of the two MAPs. Conservation of several *map*-like genes in *Bacilli *suggests involvement in a process specific to this group of organisms, which may involve also peptide deformylase.

## Methods

### Bacterial strains, plasmids, and culture conditions

*E. coli *and *B. subtilis *strains as well as plasmids used in this work are listed in Table 4. *E. coli *TG1 and XL1-Blue were used for cloning experiments (TG1 for single cross-over recombination or replicative plasmids propagation and XL1-Blue for double cross-over recombination) and *E.coli *BL5 for IPTG-induced protein overproducing. *B. subtilis *168 was used as the wild type strain in this study. *E. coli *cells were grown in Luria-Bertani (LB) medium [39] or in M63 minimal medium (KH_2_PO_4, _4.4 mM; K_2_HPO_4, _8 mM; (NH_4_)_2_SO_4_, 15 mM; MgSO_4, _2 mM; ferric citrate, 34 μM; VitB1, 0.0001% (w/v); fructose, 0.4% (w/v); sodium citrate, 0.3 mM; CaCl_2, _50 μM; MnCl_2, _5 μM; ZnCl_2, _12 μM; CuCl_2, _2.5 μM; CoCl_2, _2.5 μM; Na_2_MoO_4, _2.5 μM) supplemented with a final concentration of arabinose from 0.02% to 0.5% (w/v) when needed. *B. subtilis *cells were grown in SP medium or in minimal medium (K_2_HPO_4_, 8 mM; KH_2_PO_4_, 4,4 mM; glucose, 27 mM; Na_3_-citrate, 0.3 mM; L-glutamine, 15 mM; L-tryptophan, 0.244 mM; ferric citrate, 33.5 μM; MgSO_4 _or L-methionine, 1 mM; MgCl_2_, 0.61 mM; CaCl_2_, 49.5 μM; FeCl_3_, 49.9 μM; MnCl_2_, 5.05 μM; ZnCl_2_, 12.4 μM; CuCl_2_, 2.52 μM; CoCl_2_, 2.5 μM; Na_2_MoO_4_, 2.48 μM). Antibiotics were added to the following concentrations when required: ampicillin, 100 μg/ml; spectinomycin, 80 μg/ml; chloramphenicol, 5 μg/ml; phleomycin, 5 μg/ml; kanamycin, 100 μg/ml for *E. coli *and 5 μg/ml for *B. subtilis*. IPTG was added at 1 mM concentration or as stated when needed. Solid media were prepared by adding 1.5% agar (w/v) to the respective liquid media. Bacteria were grown at 37°C. The optical density (OD) of bacterial cultures was measured at 600 nm. All experiments were performed in accordance with the European regulation requirements concerning the contained use of Genetically Modified Organisms of Group-I (French agreement N° 2735).

### DNA techniques

DNA purification, restriction enzyme digestion, ligation and transformation of *E. coli *were performed according to standard protocols [40]. For cloning purpose, *YieldAce *or *Pfu *DNA polymerase (Stratagene) was used. All the DNA sequences in the plasmids constructed in this work were determined using the dideoxy-chain termination method and Thermo Sequenase Kit (Amersham Pharmacia Biotech). *B. subtilis *cells were transformed with plasmid DNA following the two-steps protocol described previously [41]. Transformants were selected on LB plates containing corresponding antibiotics and IPTG when needed.

### Plasmid and strain constructions

In order to clone the genes from *B. subtilis *for protein producing in *E. coli*, both *map *and *yflG *genes were amplified by PCR from genomic DNA of *B. subtilis *168 and cloned into expression vector pET24b(+) (Novagen) using *Nde*I and *Xho*I restriction sites. The amplified fragments included nucleotides +1 to +744 and nucleotides +1 to +747 relative to the translational start point of *map *and *yflG*, respectively. The resulting constructs, plasmids pIPP8003 and pIPP8004, were transformed into *E. coli *BL5 giving strains ECIP8003 and ECIP8004 for overproducing MAP and YflG, respectively.

To construct *map *and *yflG *GFP fusion plasmids and strains, both *map *and *yflG *genes were amplified by PCR from genomic DNA of *B. subtilis *168 and cloned into N-terminal GFP fusion vector pSG1729 [42] using *Xho*I and *Eco*RI restriction sites. The amplified fragments included nucleotides +1 to +744 and nucleotides +1 to +747 relative to the translational start point of *map *and *yflG*, respectively. The resulting plasmids, pIPP8001 and pIPP8002, were linearized by the *Sca*I restriction enzyme and were transformed into *B. subtilis *168. Clones were selected for the amylase deficient phenotype after double cross-over recombination at *amyE *site [42], giving strains BSIP8001 and BSIP8002, respectively.

To obtain *map *and *yflG *expression plasmids for further *map *gene rescue analysis in a conditional *map *gene deletion *E. coli *strain, EM9 [16], both *map *(nucleotides -30 to +744 relative to the *map *translation start point) and *yflG *(nucleotides -21 to +754 relative to the *yflG *translation start point) genes were amplified by PCR from genomic DNA of *B. subtilis *168 with the creation of *Bam*HI and *Bgl*II sites. Both purified PCR fragments were cleaved by *Bam*HI and *Bgl*II restriction enzymes and inserted between the same two sites in plasmid pBAD containing arabinose inducible promoter (a modified pBAD-αβγ plasmid containing an additional *Bam*HI cloning site was used after removing a *Bam*HI and *Bgl*II insert [43]), producing plasmids pHPP1017 and pHPP1018, respectively. The proper orientation was checked by the ability of cloned fragment to be recovered by double digestion using *Bam*HI and *Bgl*II restriction enzymes. Plasmids pHPP1017, pHPP1018 and the pBAD vector were transformed into *E. coli *strain EM9 individually and selected on LB plates containing ampicillin, chloramphenicol and IPTG, giving strains ECHP1005, ECHP1006 and ECHP1007, respectively.

To obtain a back-up *map *and *yflG *copies for further conditional *map *disruptant construction in *B. subtilis*, the *map *gene (nucleotides -43 to +744 relative to the *map *translation start point) and *yflG *gene (nucleotides -54 to 733 relative to the *yflG *translation start point) was amplified by PCR from genomic DNA of *B. subtilis *168 using primers introducing a *Sal*I cloning site at the 5' end and a *Sph*I cloning site at the 3' end, respectively. Both fragments were then inserted into the *Sal*I and *Sph*I sites of the IPTG-inducible replicative vector pDG148 [44], producing plasmid pHPP7021 and pHPP7022, respectively. The wild type strain *B. subtilis *168 was transformed with these plasmids, giving strains BSHP7045 and BSHP7038, respectively.

To disrupt the *map *gene, a 246 bp-long fragment within the *map *gene (nucleotides +142 to +388 relative to the *map *translation start point) was amplified by PCR from genomic DNA of *B. subtilis *168 using primers introducing an *Eco*RI cloning site at the 5' end and a *Bam*HI cloning site at the 3' end of the fragment. This fragment was then inserted into the *Eco*RI and *Bam*HI sites of plasmid pJM783 [45], which contains a promoterless *lacZ *reporter gene, producing plasmid pHPP7025. The plasmid was introduced into the chromosome of BSHP7045 and BSHP7038 strains by a single cross-over event, giving strains BSHP7046 and BSHP7037, respectively. To ascertain that they were correctly inserted, clones were checked by PCR.

To construct a *map *transcriptional fusion with the *lacZ *gene, a DNA fragment downstream from the *map *gene (nucleotides from + 423 relative to the translation start point to the gene's stop codon) was amplified by PCR from genomic DNA of *B. subtilis *168 using primers introducing an *Eco*RI cloning site at the 5' end and a *Bam*HI cloning site at the 3' end of the fragment, then inserted into the *Eco*RI and *Bam*HI sites of plasmid pJM783 [45], producing plasmid pHPP7017. The plasmid was introduced into the chromosome of *B. subtilis *168 by a single crossover event, giving strain BSHP7042.

The *yflG *transcriptional fusion with the *lacZ *gene (*yflG*::*lacZ *disruptant, strain BFS4611) was constructed within the framework of the European Union and Japanese project for the functional analysis of the genome of *B. subtilis*, where more than 2000 genes have been disrupted by fusion with *lacZ *reporter gene [22,46,47].

To construct a *yflH *transcriptional fusion with the *lacZ *gene, a DNA fragment downstream from the *yflH *gene (nucleotides from + 32 to +310 relative to the translation start point) was amplified by PCR from genomic DNA of *B. subtilis *168 using primers introducing an *Eco*RI cloning site at the 5' end and a *Bam*HI cloning site at the 3' end of the fragment, then inserted into the *Eco*RI and *Bam*HI sites of plasmid pJM783 [45], producing plasmid pHPP7018. The plasmid was introduced into the chromosome of *B. subtilis *168 by a single cross-over event, giving strain BSHP7043.

To obtain the disruptant *yvoA *strain compatible with BFS4611, the BFS817 strain (*yvoA*::*lacZ *disruptant, from the European Union and Japanese consortium, see above) was transformed with the *Sca*I linearized pEC23 plasmid (which carries a kanamycin resistance gene, M. Simon and P. Stragier, unpublished) for replacement with the kanamycin resistance gene of the *lacZ *and *Erm *genes belonging to the pMutin plasmid (erythromycin resistant) integrated into the genome, by homologous recombination. The resulting clones were checked for their inability to grow on erythromycin and chloramphenicol. The resulting strain was named BSIP8003. The chromosomal DNA of BSIP8003 was prepared and used to transform the BFS4611 strain, producing the double *yvoA *and *yflG *disrupted strain BSIP8004.

### Expression and purification of MAP_BS and YflG_Bs proteins

The strains ECIP8003 and ECIP8004 were grown at 37°C in LB medium containing kanamycin (25 μg/ml) and induced with 0.5 mM IPTG for four hours. Cell pellets were harvested by centrifugation and stored at -20°C. Cells were suspended in 20 mM Tris-HCl (pH 8.0) and sonically disrupted. The cellular debris was removed by centrifugation at 12,000 g for 40 min. The supernatants were applied to a DEAE-sephacel column chromatography (Amersham). The protein (MAP_Bs or YflG_Bs) was eluted with 0.1 M NaCl in the same buffer. Protein concentration was determined by the Bradford's method [48] using protein assay kit (Bio-Rad Laboratories). The purity of isolated proteins was determined by SDS-PAGE [40]. Ion mass spectrometry was used to measure the molecular mass of each purified protein as well as checking the existence of the first methionine in each of the proteins.

### Enzyme assays

The methionine aminopeptidase assay was performed as described by Arie Ben-Bassat et *al*. [6] with small modifications. 10 μl protein solution was added to 90 μl of the substrate solution (4 mM peptide in 0.1 M K_2_HPO_4 _(pH7.5), and 0.2 mM CoCl_2_), then incubated at 37°C for 10 min. The reaction was stopped by incubation at 100°C for 2 min. After addition of 900 μl of the colour development mixture (containing 0.2 mg of L-amino acid oxidase, 0.03 mg of horseradish peroxidase, and 0.2 mg of o-dianisidine dihydrochloride in 0.1 M Tris-HCl (pH 7.4)), the tube was incubated at 37°C for 10 min, then the absorbance at 440 nm was recorded. Standard curve with known concentration of L-methionine in the colour development buffer was plotted for quantitative analysis. The absorbance of 1 μmol of methionine per ml at 440 nm is equivalent to 8.6 [6]. One Unit of activity was defined as 1 μmol of methionine produced per min under the assay conditions used. Preference of metal ions was tested by changing the cobalt in the substrate solution into other divalent metal ions at a final concentration of 0.2 mM.

Amylase activity was detected after growth of *B. subtilis *strains on Tryptose Blood Agar Base (TBAB, Difco) supplemented with 10 g/l hydrolyzed starch (Sigma). Starch degradation was detected by sublimating iodine onto the plates [49].

β-galactosidase specific activity was measured as described by Miller [50]. One Unit of β-galactosidase activity was defined as the amount of enzyme that produced 1 nmol of *o*-nitrophenol per min at 28°C. Specific activity was expressed in Units per mg protein. The experiments were performed in triplicates.

### Subcellular localization analysis

To visualize GFP-MAP_Bs and GFP-YflG_Bs fusions locations in living cells, BSIP8001 and BSIP8002 strains were grown in minimal medium with 0.5% fructose as the sole carbon source supplemented with xylose to different final concentrations (0.1% to 1% (w/v)). Cells from the mid-exponential growth phase were collected and visualized by fluorescence microscopy. Microscope Axiovert 135 TV (ZEISS, Germany) was used in the experiment and fluorescence filter sets used to visualize GFP were obtained from Chroma Technology Corp (USA). Cells were visualized on 1% agarose (w/v) slides [51] using Axiovision 4 system (Allied High Tech Products, Inc.) with exposure time of 3–10 s.

### Genetic rescue experiment

The growth of EM9, ECHP1005 (containing *B. subtilis map *gene), ECHP1006 (containing *B. subtilis yflG *gene) and ECHP1007 (containing pBAD vector alone), was compared on M63 minimal medium plates (37°C, overnight) in the presence or absence of IPTG (1 mM) with or without arabinose (0.02 %, 0.1 % and 0.5 %).

### RNA isolation and manipulation

Total cellular RNA was extracted from *B. subtilis *168 cells growing in minimal medium to an OD_600 _of 0.5 using High Pure RNA Isolation Kit from Roche. The RNA concentration was determined by light absorption at 260 nm and 280 nm. 2 μg of RNA were loaded onto 1.2% agarose gel to check the RNA purity and integrity. RT-PCR experiments were performed using RT-PCR System (Promega) as specified by the manufacturer.

Primer extension analysis was carried out using reverse transcriptase AMV (Roche) as described [52]. Two oligonucleotides (+33 to +62 and +97 to +126 relative to translation start point of *yflG*) were used for the identification of *yflG *promoter. The same primers were used for the generation of sequence ladders. Reaction products were separated on 7% denaturing polyacrylamide gel containing 8 M urea. DNA sequences were determined using Sanger's dideoxy chain-termination method with "Thermo Sequenase radiolabeled terminator cycle sequencing kit" from Amersham Pharmacia Biotech.

### DNA sequence and *in silico *analysis

DNA sequences were analyzed using the DNA Strider software [53]. The program BLAST [54] was used to search for homologous sequences in the database. The program CLUSTALW was used for multiple alignment [55]. *B. subtilis *sequences were analyzed using the SubtiList database [56,57]. Accepted consensus sequences were extracted from PROSITE [58,59].

### Evolutionary pattern analyses

First, MAP orthologs (orthologs were defined using the BBH (Bi-directional Best Hit) strategy, i.e. if gene *a *of genome *A *is the most similar hit of gene *b *in genome *B *and vice versa, gene *a *and *b *are regarded as a pair of orthologs [60]) were retrieved between each pair of bacterial genomes. Secondly, the BBHs, with protein sequence similarity >50% and protein length difference <1.3, of the gene *map *in *B. subtilis *were collected. Thirdly, the orthologs of each of the BBHs were collected in all the genomes (a total of 206 bacterial genomes in EMBL as of July 1st, 2005), after recursive identification until the members in this cluster is no longer extended. Last, the protein divergence (sequence similarity divided by sequence length difference) of MAP in *B. subtilis *and its orthologs from other Firmicutes were compared with bacterial evolutionary distance (measured by 16S rRNA evolution). Same analyses were carried out on ClpP, Def, RpsD and YkrB.

## Authors' contributions

CHY carried out MAPs enzyme activity analysis, subcellular observation, expression level comparisons, and drafted the manuscript. HYL did genetic rescue experiment in *E. coli *and primer extension analysis. AS designed the initial experiments, completed the genetic rescue experiment in *B. subtilis*, constructed *lacZ *fusion mutants of *B. subtilis*, and wrote part of the manuscript. GF did the evolutionary pattern analyses. YPW helped to analyse the experimental data and draft manuscript. AMG supervised the MAPs enzyme activity analysis, and helped to draft the manuscript. AD designed and supervised the whole study.

## Figures and Tables

**Table 1:** Reaction rate of MAP_Bs and YflG_Bs with different peptides as the substrates

	**Specific Activity (U/mg)**	
	
**Peptide (4 mM)**	MAP_Bs	YflG_Bs
MGMM	2.6 ± 0.3	232.0 ± 8.2
MAS	2.0 ± 0.1	41.0 ± 5.5
MG	1.2 ± 0.1	1.3 ± 0.1

Three measurements for each of the proteins with different substrates were made.

**Table 2:** MAP activity in crude cell extracts of *E. coli *and *B. subtilis*

	**Specific Activity (U/mg)**		
	
**Peptide (4 mM)**	*E. coli *(BL5)	*B. subtilis *wild type (168)	*B. subtilis yflG *mutant (BFS4611)
MGMM	1.5 ± 0.2	1.5 ± 0.1	1.5 ± 0.1
MAS	1.1 ± 0.2	1.1 ± 0.1	1.1 ± 0.2
MG	0.6 ± 0.1	0.8 ± 0.2	0.4 ± 0.1

Cells in stationary growth phase of each of the strains were collected and sonically disrupted. Supernatants of each of the cell debris were used to measure MAP activity. Totally, three times of measurements were carried out.

**Table 3:** Distribution of *def *and *map *genes in selected genomes

	**Numbers in Each Genome**		
		
**Genome Name**	***def***	***map***	**EMBL ID**
*Streptomyces coelicolor*	4	3	AL645882
*Streptomyces avermitilis*	3	4	BA000030
*Nocardia farcinica IFM 10152*	3	3	AP006618
*Shewanella oneidensis MR-1*	3	1	AE014299
*Silicibacter pomeroyi DSS-3*	3	1	CP000031
*Bacillus cereus ZK*	2	3	CP000001
*Bacillus thuringiensis serovar konkukian*	2	3	AE017355
*Bacillus anthracis 'Ames Ancestor'*	2	3	AE017334
*Bacillus anthracis Sterne*	2	3	AE017225
*Bacillus cereus ATCC 10987*	2	3	AE017194
*Bacillus anthracis Ames*	2	3	AE016879
*Bacillus cereus ATCC 14579*	2	3	AE016877
*Leifsonia xyli subxyli CTCB07*	2	3	AE016822
*Synechocystis PCC 6803*	1	3	BA000022
*Bacillus clausii KSM-K16*	1	3	AP006627

**Table 4:** Bacterial strains and plasmids used or created in this study

**Strains**	**Genotype**	**Source or Reference**
*Escherichia coli*		

XL1-Blue	K12 *supE*44 *hsdR*17 *recA*1 *endA*1 *gyrA*46 *thi relA*1 *lac*^- ^F'[*proAB*^+ ^*lacI*^q ^*lacZΔM*15 Tn10(tet^R^)]	Laboratory collection
TG1	K12 *supE hsdΔ*5 *thi Δ *(*lac-proAB*) F'[*traD*36 *proA*^+ ^*proB*^+ ^*lacI*^q ^*lacZ*Δ*M*15]	Laboratory collection
BL5(DE3)	F^-^	Invitrogen
EM9	P_T7_-*map *(on chromosome)	Chiron Culture Collection Centre
ECIP8003	F^- ^pIPP8003	This work
ECIP8004	F^- ^pIPP8004	This work
ECHP1005	EM9 pHPP1017	This work
ECHP1006	EM9 pHPP1018	This work
ECHP1007	EM9 pBAD	This work

*Bacillus subtilis*		

168	*trp*C2	Laboratory collection
BFS4611	*trp*C2 *yflG*::*lacZ*	Functional analysis project^a^
BFS817	*trp*C2 *yvoA*::*lacZ*	Functional analysis project^a^
BSHP7042	*trp*C2 *map-lacZ*	This work
BSHP7045	*trp*C2 pDG148-*map*	This work
BSHP7046	*trp*C2 *map*::*lacZ *pDG148-*map*	This work
BSHP7037	*trp*C2 *map*::*lacZ *pDG148-*yflG*	This work
BSHP7038	*trp*C2 pDG148-*yflG*	This work
BSHP7043	*trp*C2 *yflH*::*lacZ*	This work
BSIP8001	*trp*C2 *amyE*::*gfp-map*	This work
BSIP8002	*trp*C2 *amyE*::*gfp-yflG*	This work
BSIP8003	*trp*C2 *yvoA*::[(*lacZ*-ery)::pEC23(Kan)]	This work
BSIP8004	*trp*C2 *yflG*::*lacZ yvoA*::[(*lacZ*-ery)::pEC23(Kan)]	This work

**Plasmids**	**Description**	**Source or Reference**

pET24b(+)	expression vector, Kan^R^	Novagen
pBAD-abg	genetic rescue vector, Amp^R^	[43]
pDG148	expression vector, Amp^R ^Kan^R^	[44]
pJM783	cloning vector, Cm^R^	[45]
pSG1729	N-terminal GFP fusion vector, Amp^R ^Spec^R^	[42]
pEC23	integrative plasmid, Kan^R^	M. Simon and P. Stragier, unpublished
pIPP8003	pET24b *map*	This work
pIPP8004	pET24b *yflG*	This work
pHPP1017	pBAD *map*	This work
pHPP1018	pBAD *yflG*	This work
pHPP7017	pJM783 *map-lacZ*	This work
pHPP7018	pJM783 *yflH*::*lacZ*	This work
pHPP7021	pDG148 *map*	This work
pHPP7022	pDG148 *yflG*	This work
pHPP7025	pJM783 *map*::*lacZ*	This work
pIPP8001	pSG1729 *map*	This work
pIPP8002	pSG1729 *yflG*	This work

^a ^This strain has been constructed during the frame of the project for the functional characterization of the genome of *B. subtilis *in Japan [46].
